# Total hysterectomy versus radical hysterectomy in neuroendocrine cervical cancer: a SEER-database analysis

**DOI:** 10.1007/s00432-024-05773-8

**Published:** 2024-05-06

**Authors:** Feitianzhi Zeng, Peng Guo, Meng Xia, Mian He

**Affiliations:** Department of Gynecology, The First Affiliated Hospital of Sun Yat-Sen University, Guangzhou, Guangdong China

**Keywords:** Neuroendocrine cervical cancer, Radical hysterectomy, Total hysterectomy

## Abstract

**Purpose:**

We conducted this study to evaluate the efficacy of total hysterectomy versus radical hysterectomy in the treatment of neuroendocrine cervical cancer (NECC).

**Methods:**

Eligible NECC patients were identified from the Surveillance, Epidemiology and End Results (SEER) database. Demographic characteristics, clinical treatment and survival of the patients were collected. The overall survival (OS) and cancer-specific survival (CSS) were estimated by Kaplan–Meier analysis with log-rank test.

**Results:**

A total of 286 patients were included, with 104 patients undergoing total hysterectomy and 182 patients undergoing radical hysterectomy. The 5-year OS were 50.8% in the total hysterectomy group and 47.5% in the radical hysterectomy group (p = 0.450); and the corresponding 5-year CSS were 51.6% and 49.1% (p = 0.494), respectively. Along with surgery, radiotherapy was given to 49.0% of patients in the total hysterectomy group and 50.5% in the radical hysterectomy group; and chemotherapy was administered to 77.9% of patients in the total hysterectomy group and 85.7% in the radical hysterectomy group. Unexpectedly, in patients who received adjuvant radiotherapy with or without chemotherapy, the OS was superior in the total hysterectomy group compared with the radical hysterectomy group (p = 0.034). While in patients who received chemotherapy alone and those who received neither radiotherapy nor chemotherapy, the OS still remained comparable between the total hysterectomy and radical hysterectomy group.

**Conclusion:**

Compared with radical hysterectomy, total hysterectomy was not associated with compromised survival prognosis in patients with NECC. Total hysterectomy has the potential to be a surgical alternative in the multimodal management of NECC.

**Supplementary Information:**

The online version contains supplementary material available at 10.1007/s00432-024-05773-8.

## Introduction

NECC is a rare type of cervical malignancy, comprising 1–1.5% of all cases(Tempfer et al. 2018). Due to its aggressive biological behavior, NECC often leads to lymph node and widespread hematogenous metastasis. Previous studies have indicated that 31.5–49.5% of NECC patients had lymph nodal involvement, and 4–13% had distant metastasis at initial diagnosis(Cohen et al. 2010; Stecklein et al. 2016; Castelnau-Marchand et al. 2018; Zhang et al. 2019). The prognosis for NECC patients remains poor even in the early stage, with a 5-year survival rate of only 31.6–36.8%, compared with up to 87% for common cervical cancer(Chan et al. 2003; Cohen et al. 2010, 2019).

Radical hysterectomy with regional lymphadenectomy is recommended as a primary treatment option for patients with early-stage NECC(Gardner et al. 2011; Satoh et al. 2014; National Comprehensive Cancer Network 2022). Compared with total hysterectomy, radical hysterectomy is characterized by extended resection of the parametrium, upper vagina and partial uterosacral ligaments. The purpose of radical hysterectomy for cervical cancer includes removing potential micro-metastasis in the soft tissue surrounding the cervix to prevent pelvic recurrence, and providing pathological information of the parametrium as an indicator for adjuvant treatment(Wright et al. 2007). However, due to the clinical aggressiveness of NECC, adjuvant radiation and chemotherapy are almost routinely considered for NECC patients, regardless of their pathological risk factors(Salvo et al. 2019; National Comprehensive Cancer Network 2022). Therefore, the question whether radical hysterectomy is necessary for NECC patients has been raised(Salvo et al. 2021).

On the other hand, in comparison to total hysterectomy, radical hysterectomy has a higher risk of surgery related complications, such as urinary, nerve and vascular injury(Obermair et al. 2020; Kim et al. 2021; Plante et al. 2023). The incidence of postoperative urinary incontinence and urinary retention is 11.0% and 9.9%, respectively, in patients receiving radical hysterectomy, compared to 4.7% and 0.6%, respectively, in patients undergoing total hysterectomy(Plante et al. 2023). Thus, avoidance of radical hysterectomy may reduce surgical morbidity and improve postoperative quality of life.

Notwithstanding, it remains unclear whether the substitution of total hysterectomy for radical hysterectomy in the multimodal treatment of NECC would compromise the survival prognosis of the patients. Therefore, we conducted this retrospective study to compare the efficacy of total hysterectomy and radical hysterectomy in the treatment of NECC using data from the SEER database.

## Methods

### Patients selection

The research data for this study was obtained from the SEER database, using the SEER*Stat software (version 8.4.0; National Cancer Institute, USA). According to the International Classification of Diseases for Oncology, Third Edition (ICD-O-3), we identified patients with NECC using the primary site codes of C53.0-C53.1, C53.8-C53.9 and the histology codes of 8012, 8013, 8014, 8041, 8043, 8240, 8246 and 8249. Patients diagnosed between 1998 and 2017, aged 20–84 years, and treated with total or radical hysterectomy were eligible for this study. Patients with unavailable survival data were excluded.

### Data collection

Demographic and clinical characteristics of the patients were collected, including age at diagnosis, histology, stage, tumor size, lymph node status, distant metastasis, surgery of primary site, radiotherapy, chemotherapy and survival status. The primary endpoints of this study were OS and CSS, which were defined as the time interval from diagnosis to death from any cause and from cancer, respectively.

### Statistical analysis

The t-test was used to compare the differences in continuous variables, and the χ^2^ test or Fisher’s exact test was used for categorial variables. OS and CSS were estimated by the Kaplan–Meier analysis with log-rank test. The Cox regression analysis was used for multivariate analysis to estimate the effect of potential prognostic factors. P-value < 0.05 was considered as statistically significant. The IBM SPSS Statistics for Windows, version 26.0 (IBM Corp, Armonk, NY) and GraphPad Prism version 5.0 for Windows (GraphPad Software, San Diego, CA) were used for statistical analysis.

## Results

### Demographic characteristics

A total of 286 patients who met the inclusion criteria were identified from the SEER database. Among them, 104 patients underwent total hysterectomy and the other 182 patients underwent radical hysterectomy. Demographic characteristics of patients in both groups, including age at diagnosis, histology, tumor size, stage, lymph node status, and distant metastasis, were summarized in Table 1. The proportion of patients who diagnosed at age 45 years or older was significantly higher in the total hysterectomy group than in the radical hysterectomy group (55.8% vs 36.3%, p = 0.002). Regarding lymph node metastasis, 36.6% and 31.7% of patients in the total hysterectomy group were found to be negative and with unknown status, respectively, while the corresponding ratios in the radical hysterectomy group were 56.0% and 7.7% (p < 0.001). All other demographic characteristics were comparable between the total hysterectomy group and the radical hysterectomy group.

**Table 1:** Demographic characteristics of patients

	Total hysterectomy (*n* = 104)		Radical hysterectomy (*n* = 182)		*P*
	n	%	n	%	
Age					0.002
< 45	46	44.2	116	63.7	
≥ 45	58	55.8	66	36.3	
Histology					0.187
Small cell carcinoma	65	62.5	97	53.3	
Large cell carcinoma	12	11.5	31	17.0	
Carcinoid	1	1.0	0	0.0	
NOS	26	25.0	54	29.7	
Tumor size					0.236
≤ 4 cm	50	48.1	103	56.6	
> 4 cm	29	27.9	49	26.9	
Unknown	25	24.0	30	16.5	
FIGO stage					0.216
I	63	60.6	127	69.8	
II	13	12.5	25	13.7	
III	3	2.9	6	3.3	
IV	23	22.1	22	12.1	
Unknown	2	1.9	2	1.1	
Lymph node metastasis					< 0.001
No	38	36.6	102	56.0	
Yes	33	31.7	66	36.3	
Unknown	33	31.7	14	7.7	
Distant metastasis					0.113
No	82	78.8	159	87.4	
Yes	21	20.2	22	12.1	
Unknown	1	1.0	1	0.5	

*NOS* not otherwise specified, *FIGO* International Federation of Gynecology and Obstetrics

### Treatments

The treatments administered to patients in each group were presented in Table 2, including surgery for regional lymph node and distant metastatic lesion, radiotherapy, and chemotherapy.

**Table 2:** Treatment details of patients

	Total hysterectomy (*n* = 104)		Radical hysterectomy (*n* = 182)		P
	n	%	n	%	
Regional LN surgery					< 0.001
Resection	62	59.6	135	74.2	
Biopsy	5	4.8	32	17.6	
None	36	34.6	13	7.1	
Unknown	1	1.0	2	1.1	
Distant site surgery					0.635
Resection	5	4.8	6	3.3	
Non-primary surgical procedure	6	5.8	8	4.4	
None/Biopsy	93	89.4	168	92.3	
Radiotherapy					0.902
Yes	51	49.0	92	50.5	
None/Unknown	53	51.0	90	49.5	
Form of radiotherapy					0.023
Beam radiation	26	51.0	65	70.7	
Beam radiation + brachytherapy	21	41.2	22	23.9	
Brachytherapy	2	3.9	5	5.4	
NOS	2	3.9	0	0.0	
Sequence of radiotherapy					0.002
Radiation after surgery	34	66.7	83	90.2	
Radiation prior to surgery	14	27.4	7	7.6	
Radiation before and after surgery	1	2.0	1	1.1	
Sequence unknown	2	3.9	1	1.1	
Chemotherapy					0.104
Yes	81	77.9	156	85.7	
None/unknown	23	22.1	26	14.3	
Sequence of chemotherapy					0.061
Chemotherapy after surgery	37	45.7	90	57.7	
Chemotherapy prior to surgery	12	14.8	8	5.1	
Chemotherapy before and after surgery	5	6.2	8	5.1	
Sequence unknown	27	33.3	50	32.1	

*LN* lymph node, *NOS* not otherwise specified

74.2% of patients in the radical hysterectomy group received regional lymph node resection and 17.6% received lymph node biopsy. Whereas in the total hysterectomy group, the corresponding ratios were 59.6% and 4.8%, respectively. In both groups, fewer than 5% of patients had their distant metastatic lesions resected. Radiotherapy was given to 49.0% of patients in the total hysterectomy group and 50.5% in the radical hysterectomy group. In patients who had radiotherapy, 92.2% in the total hysterectomy group and 94.6% in the radical hysterectomy group received external beam radiation with or without brachytherapy. Chemotherapy was administered to 77.9% of patients in the total hysterectomy group and 85.7% in the radical hysterectomy group. However, information on the chemotherapy regimen and number of chemotherapy cycles were not available in the database.

Furthermore, patients in the total hysterectomy group were found to receive neoadjuvant therapy more often than those in the radical hysterectomy group. 29.4% of patients in the total hysterectomy group underwent preoperative radiotherapy and 21.0% of them underwent preoperative chemotherapy, both higher than the corresponding rates in the radical hysterectomy group of 8.7% (p = 0.002) and 10.2% (p = 0.061), respectively.

### Survival analysis

**Fig. 1:**
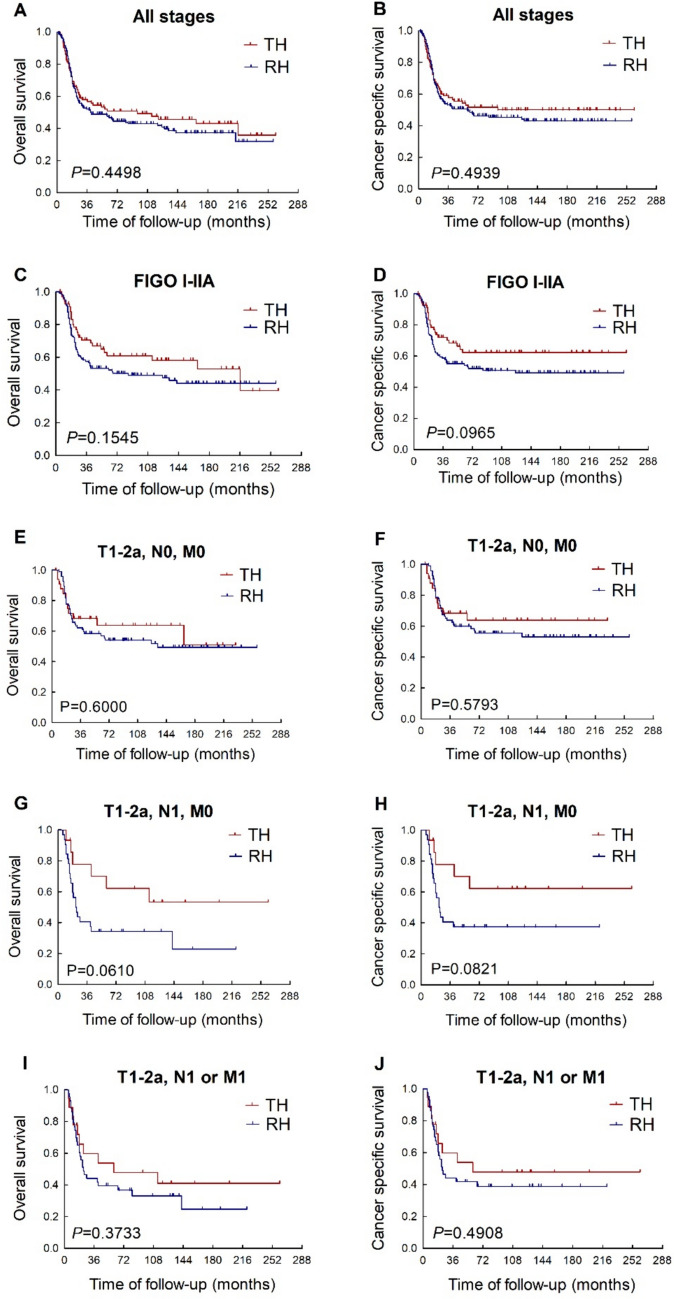
Comparison of total hysterectomy and radical hysterectomy in NECC patients in different stages. *TH* total hysterectomy. *RH*: radical hysterectomy

The 5-year OS rate were 50.8% in the total hysterectomy group and 47.5% in the radical hysterectomy group (Fig. 1A); and the corresponding 5-year CSS rate were 51.6% and 49.1%, respectively (Fig. 1B). There’s no statistically significant difference in either OS (*P* = 0.450; HR, 0.881; 95% CI, 0.633–1.227) or CSS (*P* = 0.494; HR, 0.887; 95% CI, 0.627–1.255) between the two groups.

Patients with FIGO stage I-IIA cervical cancer are considered as candidates for radical surgery according to most clinical guidelines. In the subset of patients with FIGO stage I-IIA NECC (Fig. 1C, D), it’s unexpected that the survival prognosis appeared to be better in the total hysterectomy group than in the radical hysterectomy group, although the difference in both OS (p = 0.154; HR, 0.735; 95% CI, 0.482–1.123) and CSS (p = 0.096; HR, 0.686; 95% CI, 0.440–1.070) between the total hysterectomy group and the radical hysterectomy group didn’t reach statistical significance.

To investigate the impact of lymph node status, we further divided patients with FIGO stage I-IIA NECC into two subsets, namely patients with lymph node metastasis (T1-2a/N1/M0) and without lymph node metastasis (T1-2a/N0/M0). In the subset of T1-2a/N0/M0 patients (Fig. 1E, F), both OS (p = 0.600; HR, 0.848; 95% CI, 0.458–1.571) and CSS (p = 0.579; HR, 0.834; 95% CI, 0.440–1.583) were comparable between the total hysterectomy group and the radical hysterectomy group. While in the subset of T1-2a/N1/M0 patients (Fig. 1G, H), the superiority of survival prognosis in patients underwent total hysterectomy to those underwent radical hysterectomy seemed to be further improved, but the difference in both OS (p = 0.061; HR, 0.475; 95% CI, 0.218–1.035) and CSS (p = 0.082; HR, 0.480; 95% CI, 0.210–1.098) between these two groups still didn’t reach statistical significance.

Due to the aggressiveness of NECC, patients may present with distant metastatic lesions while the primary lesion remains confined to the cervix and upper vagina (T1-2a/N1 or M1), which is considered as resectable. In the subset of patients with T1-2a/N1 or M1 disease (Fig. 1I, J, the OS (p = 0.373; HR, 0.734; 95% CI, 0.371–1.450) and CSS (p = 0.491; HR, 0.775; 95% CI, 0.376–1.599) were still comparable between the total hysterectomy group and the radical hysterectomy group.

A multivariate Cox regression was conducted to assess the impact of potential prognostic factors, including age, lymph node status, FIGO stage, main surgery type, radiotherapy, and chemotherapy. The analysis revealed that age older than 45, lymph node metastasis and advanced stage were identified as risk factors for both OS and CSS (Supplementary Table 1).

### The effects of adjuvant treatments

**Fig. 2:**
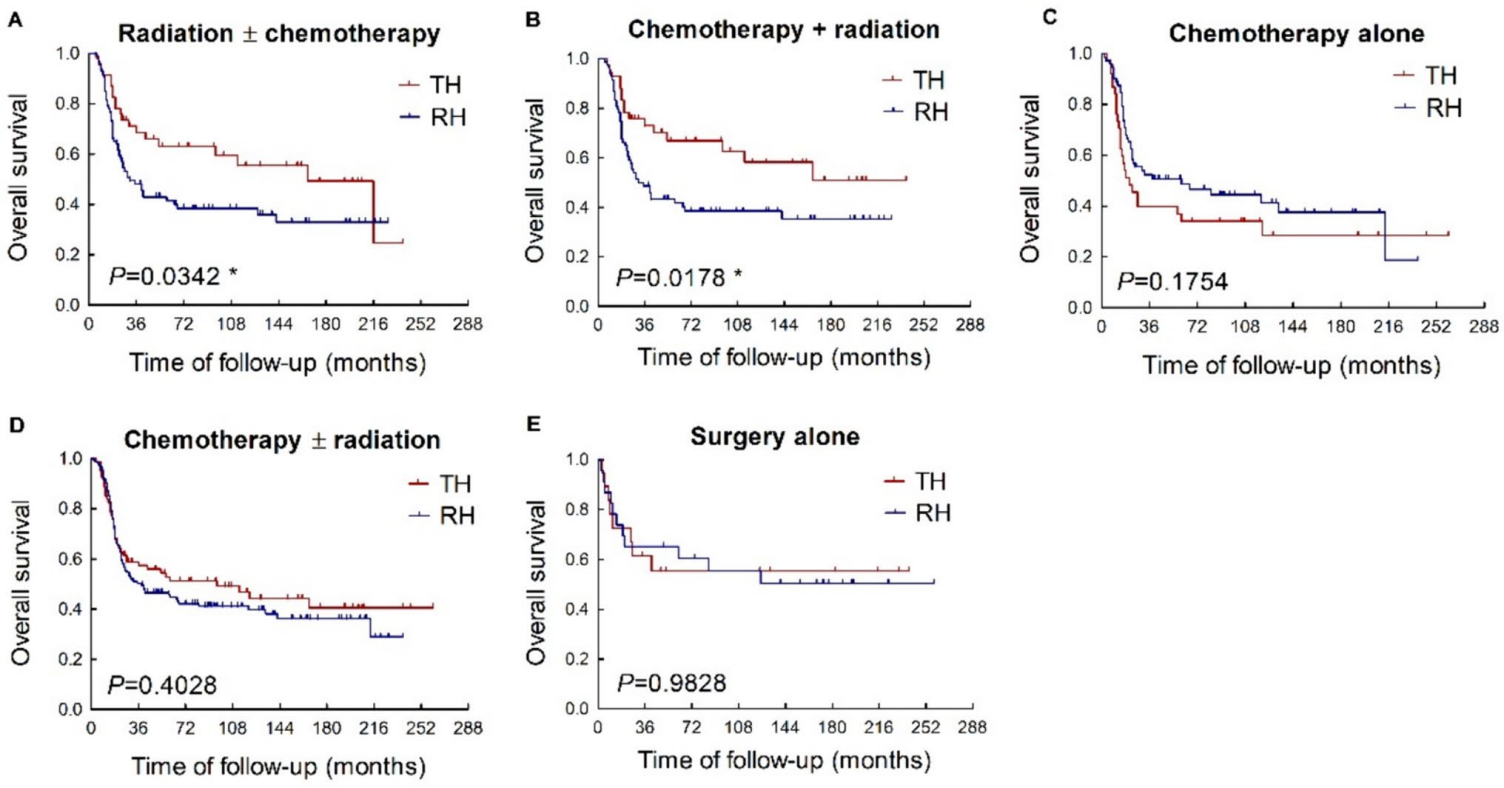
Comparison of total hysterectomy and radical hysterectomy in NECC patients receiving different adjuvant therapies. *TH*: total hysterectomy. *RH*: radical hysterectomy

Adjuvant therapy was given to 85.3% of patients in this study. Among them, 127 patients received both adjuvant chemotherapy and radiotherapy, 110 patients received adjuvant chemotherapy alone and 7 patients received adjuvant radiotherapy alone. The addition of adjuvant radiotherapy with or without chemotherapy (Fig. 2A) yielded significantly better OS (p = 0.034; HR, 0.598; 95% CI, 0.372–0.963) in the total hysterectomy group compared with the radical hysterectomy group. Similar result was found in patients receiving both adjuvant radiotherapy and chemotherapy (Fig. 2B). However, there was no statistically significant difference in OS between the total hysterectomy and radical hysterectomy groups among patients who received adjuvant chemotherapy alone (Fig. 2C) or adjuvant chemotherapy with or without radiotherapy (Fig. 2D). The OS in patients who received neither adjuvant radiotherapy nor chemotherapy (Fig. 2E) was also comparable between the two groups.

Similar results were also observed in the subgroup of patients with FIGO stage I-IIA NECC. When adjuvant radiotherapy with or without chemotherapy was administered, the patients undergoing total hysterectomy exhibited significantly improved CSS compared to those undergoing radical surgery (Supplementary Fig. 1C; p = 0.047; HR, 0.552; 95% CI, 0.307–0.993). While in patients who received adjuvant chemotherapy alone or no adjuvant treatment, CSS remained comparable between the total and radical hysterectomy groups (Supplementary Fig. 1A, D).

**Fig. 3:**
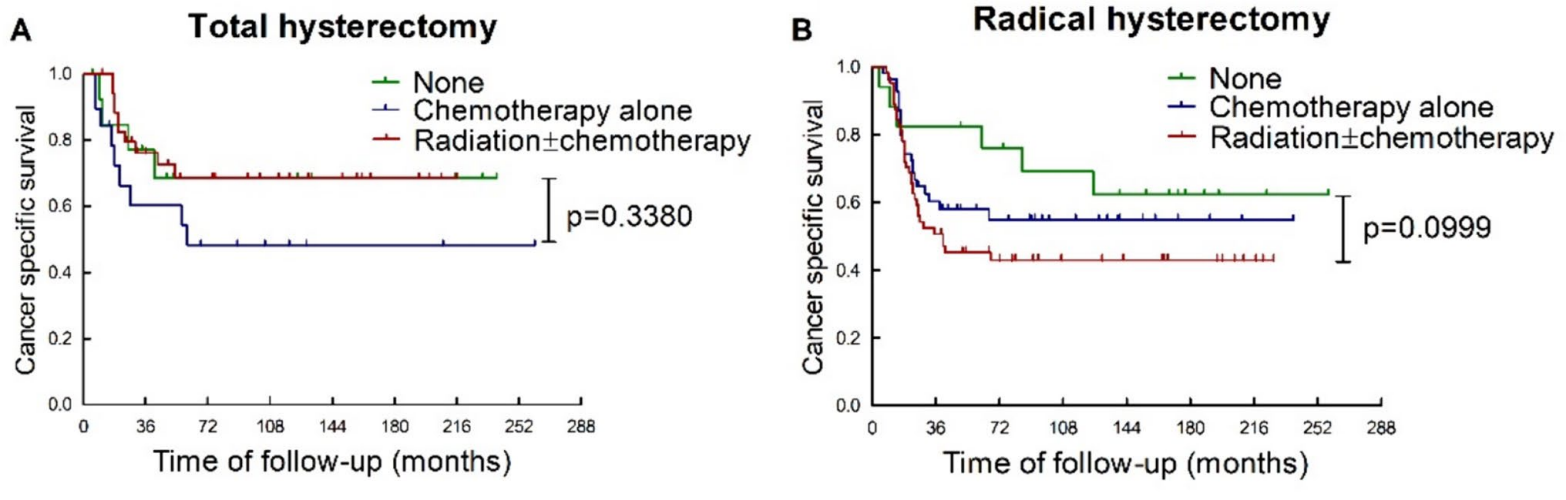
Influence of adjuvant therapies on the efficacy of total hysterectomy and radical hysterectomy in patients with FIGO stage I-IIA NECC

Furthermore, our study showed that patients in the radical hysterectomy group were less likely to benefit from adjuvant treatments. In the total hysterectomy group (Fig. 3A), the CSS in patients with FIGO stage I-IIA NECC was comparable between those who received adjuvant radiotherapy and those who didn’t receive any form of adjuvant treatment. While in the radical hysterectomy group (Fig. 3B), the CSS in patients with FIGO stage I-IIA NECC was deteriorated when adjuvant radiotherapy and chemotherapy was administered, although statistical significance was not reached.

## Discussion

In this study, we found that total hysterectomy didn’t compromise the OS or CSS in patients with NECC compared with radical hysterectomy. Furthermore, our study showed that when adjuvant radiotherapy was administered, total hysterectomy could even provide a better survival prognosis than radical hysterectomy in NECC patients.

In the NeCTuR study that evaluated 100 patients with early-stage NECC who underwent upfront radical surgery, 95% of patients met the criteria for adjuvant therapy based on pathological risk factors (Salvo et al. 2021). Parametrial involvement was found in 10% of patients in the NeCTuR study, while all of these patients had other coexisting indications for adjuvant therapy (Salvo et al. 2021). Given the poor prognosis of NECC, most clinical guidelines recommend the combination of surgery, chemotherapy and radiotherapy as a multimodal management, regardless of pathological risk factors (Salvo et al. 2019; National Comprehensive Cancer Network 2022). Therefore, the question has been raised whether radical surgery can be replaced by simple hysterectomy in the multimodal treatment of NECC to minimize postoperative morbidity and improve quality of life.

The finding in our study demonstrated that the efficacy of total hysterectomy might not be inferior to radical hysterectomy in NECC. It could be explained by the fact that early-stage patients might already have distant micro-metastasis, due to the aggressive nature of NECC. In our study, 21% of the patients presented with positive lymph nodes and/or distant metastasis while their cervical lesions were still in the early stages. As shown in the ABRAX trial, completion of radical hysterectomy didn’t improve the survival compared with definitive chemoradiation in cervical cancer patients with lymph node metastasis, regardless of histological type (Cibula et al. 2021). Removal of the primary cervical lesion and the potential regional metastatic lesion by radical hysterectomy may not be efficient enough if there has been distant micro-metastasis.

Besides, the recurrence pattern may also provide an explanation for why patients with NECC benefit less from radical hysterectomy. Previous studies have shown that 60–70% of NECC patients experience a recurrence outside the pelvis, with the lung and liver being the most common sites of recurrence (Stecklein et al. 2016; Ishikawa et al. 2018; Salvo et al. 2021; Pan et al. 2022). Radical hysterectomy is mainly aimed at preventing pelvic recurrence. Therefore, extensive resection of the local lesion may not be the primary prognostic factor for recurrence of NECC. Moreover, theoretically, there’s a greater chance that cancer cells will disseminate from the primary cervical lesion during a radical surgery, due to the longer duration of operation and the larger extent of resection (Suhail et al. 2019). Nevertheless, it remains unclear whether radical hysterectomy is associated with an increased risk of distant recurrence in NECC in our study, since data on the pattern of recurrence is not available from the SEER database.

Adjuvant radiotherapy is used as a local treatment to prevent pelvic recurrence and control positive regional lymph nodes in common cervical cancer patients with postoperative pathological high risk factors, including lymph node involvement, parametrium metastasis and positive surgical margins (National Comprehensive Cancer Network 2022). However, the role of adjuvant radiotherapy in NECC is controversial. A meta-analysis by Kim et al. showed that patients with early-stage NECC who received radical surgery with postoperative radiotherapy had lower pelvic recurrence rate than those who didn’t receive radiotherapy (12.5% vs 24.3%, p = 0.09), but the OS rate was not improved by postoperative radiotherapy (34.8% vs 35.2%, p = 0.66), because of the high incidence of distant recurrence in both groups (Kim et al. 2023). Kim’s study also found that the rate of distant recurrence was significantly higher in patients who received postoperative radiotherapy (33.3% vs 9.2%, *p* = 0.007), although the mechanism remains unclear (Kim et al. 2023). On the other hand, a SEER-based study by Dong et al. found that surgery with adjuvant radiotherapy was associated with significantly improved OS in NECC patients with metastasis comparing with surgery alone (median OS: 80.9 months vs 44.6 months, p = 0.004) (Dong et al. 2021). This finding indicated that NECC patients with distant metastasis may still potentially benefit from intensive local treatments containing adjuvant radiotherapy. In our study, when adjuvant radiotherapy was given, patients who underwent total hysterectomy had better survival than those who had radical hysterectomy. This intriguing finding may also be associated with the ineffectiveness of adjuvant radiation in preventing distant recurrence.

Chemotherapy is a common systematic treatment for NECC. The most widely used regimen for NECC is platinum combined with etoposide (Sundstrøm et al. 2002). The study of Seino et al. demonstrated that in patients with stage IB2 and T1N1M0 NECC, those who received surgery followed by chemotherapy had superior OS compared with those who received surgery followed by radiotherapy (Seino et al. 2023). Ishikawa’s study also showed that adjuvant chemotherapy after surgery could reduce the risk of recurrence in patients with stage I-II NECC (Ishikawa et al. 2018). In addition, previous studies have highlighted the importance of the number of adjuvant chemotherapy cycles as a prognostic factor, with patients receiving four or more cycles of adjuvant chemotherapy having significantly improved survival prognosis compared to those receiving fewer cycles (Ishikawa et al. 2019; Wang et al. 2022). In our study, over 80% of NECC patients had received chemotherapy, but adjuvant chemotherapy was not associated with improved OS, irrespective of whether they underwent total hysterectomy or radical hysterectomy. Nonetheless, we’re not able to further investigate the effect of chemotherapy on NECC in this study, because the detailed information on chemotherapy, such as regimens and number of cycles, was not available in the SEER database.

NECC is known to have a poor prognosis. Our study showed that age older than 45, lymph node metastasis and advanced stage were associated with worse survival prognosis in NECC patients. Several recent studies have also sought to identify potential prognostic factors and to construct prognostic models to improve the prediction of the oncological outcomes for NECC patients (Chen et al. 2023; Yu et al. 2024). Yu’s study identified larger tumor size, metastasis, advanced tumor stages, and chemotherapy as independent prognostic factors of OS (Yu et al. 2024). Yu et al. subsequently constructed a prognostic nomogram based on these four factors, demonstrating a C-index of 0.724, an area under the receiver operating characteristic curve (AUC) of 0.769 and 0.766 for the prediction of 3-year and 5-year OS, respectively(Yu et al. 2024). The construction of prognostic prediction models has the potential to facilitate the identification of NECC patients with high risk and the development of individualized treatment strategy.

To the best of our knowledge, our study is the first to compare the clinical outcomes of total hysterectomy and radical hysterectomy in patients with NECC. Our study has enrolled 286 NECC cases from the SEER database, the sample size of which is larger than most previous clinical studies on NECC. The results of our study indicate that total hysterectomy may be a potential surgical option in the multimodal management of NECC. However, due to the methodology and retrospective nature of this study, it has several limitations. We’re not able to compare the effect of total hysterectomy and radical hysterectomy on progression-free survival in this study, since the data of recurrence are not available in the database. Similarly, the detailed information of chemotherapy and radiotherapy, such as chemotherapy regimen, radiation therapy technique and dose, are not recorded in the database either. Non-standardized adjuvant treatment would also result in bias in this study. Therefore, future multicenter studies are needed to further evaluate the role of total hysterectomy in NECC.

## Conclusion

Our study showed that the survival prognosis in patients with NECC receiving total hysterectomy was not inferior to those undergoing radical hysterectomy, particularly when combined with adjuvant radiotherapy. Total hysterectomy may be considered as a surgical alternative in the multimodal management of NECC.

### Supplementary Information

Below is the link to the electronic supplementary material.

Supplementary file1 (DOCX 187 KB)

## Data Availability

The research data for this study was available in the SEER database: https://seer.cancer.gov/.
