# Supplementary material for: Total hysterectomy versus radical hysterectomy in neuroendocrine cervical cancer: a SEER-database analysis
Source: J Cancer Res Clin Oncol. 2024 May 6;150(5):236. doi: 10.1007/s00432-024-05773-8 (PMC11074048; doi:10.1007/s00432-024-05773-8)
Supplement: Supplementary file 1 — Supplementary file1 (DOCX 187 KB) [file 432_2024_5773_MOESM1_ESM.docx]

**Supplementary Figure 1**. Comparison of total hysterectomy and radical hysterectomy in stage I-IIA NECC patients with and without adjuvant therapies. TH: total hysterectomy. RH: radical hysterectomy


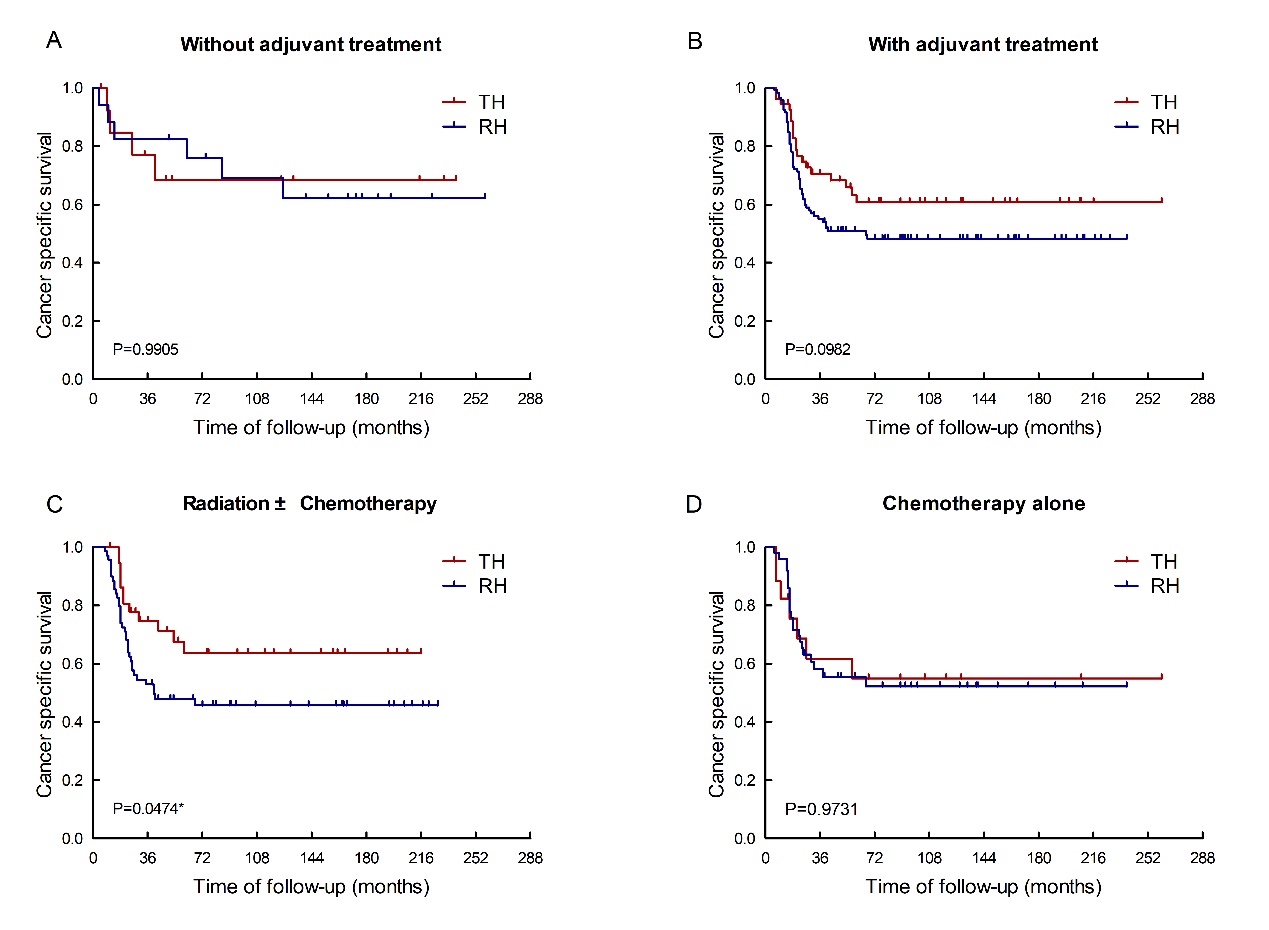


**Supplementary Table 1.** Multivariate analysis of prognostic factors

| **Variables** | **OS** | | | |  | **CSS** | | | |
| --- | --- | --- | --- | --- | --- | --- | --- | --- | --- |
|  | **HR** | **95% CI** | | ***P*** |  | **HR** | **95% CI** | | ***P*** |
| **Age** |  |  |  |  |  |  |  |  |  |
| <45 | Ref |  |  |  |  | Ref |  |  |  |
| ≥45 | 1.656 | 1.196 | 2.293 | 0.002 |  | 1.460 | 1.038 | 2.052 | 0.030 |
| **Lymph node status** |  |  |  |  |  |  |  |  |  |
| Negative | Ref |  |  | 0.001 |  | Ref |  |  | 0.001 |
| Positive | 2.116 | 1.430 | 3.132 | 0.000 |  | 2.120 | 1.407 | 3.195 | 0.000 |
| Unknown | 1.233 | 0.710 | 2.141 | 0.457 |  | 1.292 | 0.725 | 2.301 | 0.385 |
| **FIGO stage** |  |  |  |  |  |  |  |  |  |
| I-IIA | Ref |  |  | 0.032 |  | Ref |  |  | 0.029 |
| IIB-IV | 1.637 | 1.132 | 2.369 | 0.009 |  | 1.683 | 1.145 | 2.474 | 0.008 |
| Unknown | 1.329 | 0.310 | 5.698 | 0.702 |  | 1.432 | 0.332 | 6.177 | 0.631 |
| **Surgery Type** |  |  |  |  |  |  |  |  |  |
| Total hysterectomy | Ref |  |  |  |  | Ref |  |  |  |
| Radical hysterectomy | 1.277 | 0.889 | 1.834 | 0.185 |  | 1.255 | 0.858 | 1.835 | 0.241 |
| **Beam Radiation** |  |  |  |  |  |  |  |  |  |
| No/unknown | Ref |  |  |  |  | Ref |  |  |  |
| Yes | 0.894 | 0.631 | 1.266 | 0.527 |  | 0.873 | 0.606 | 1.258 | 0.466 |
| **Chemotherapy** |  |  |  |  |  |  |  |  |  |
| No/unknown | Ref |  |  |  |  | Ref |  |  |  |
| Yes | 0.912 | 0.575 | 1.446 | 0.695 |  | 0.929 | 0.571 | 1.514 | 0.769 |
